# Evaluation of lower limb kinetics during gait, sprint and hop tests before and after anterior cruciate ligament reconstruction

**DOI:** 10.1007/s10195-017-0456-9

**Published:** 2017-03-30

**Authors:** Joaquín Moya-Angeler, Javier Vaquero, Francisco Forriol

**Affiliations:** 1 Hospital Special Surgery, 535 E 70th St, New York City, NY 10021 USA; 2 University Hospital Gregorio Marañon, Calle del Dr. Esquerdo, 46, 28007 Madrid, Spain; 3 School of Medicine, University San Pablo-CEU, Madrid, Urbanización Montepríncipe, Boadilla del Monte, 28668 Madrid, Spain; 4 144 E 74th St 3r, New York, NY 10021 USA

**Keywords:** Knee kinetics, ACL deficiency, ACL reconstruction, Hop tests, Semitendinosus gracilis autograft

## Abstract

**Background:**

The purpose of this study was to evaluate the functional status prior to and at different times after anterior cruciate ligament reconstruction (ACLR), and to analyze the changes in the kinetic patterns of the involved and uninvolved lower limb during gait, sprint and three hop tests.

**Materials and methods:**

Seventy-four male patients with an ACL injury were included in the study. All patients performed a standardized kinetic protocol including gait, sprint and three hop tests (single-leg hop, drop vertical jump and vertical jump tests), preoperatively and at 3, 6, and 12 months after ACLR with a semitendinosus gracilis tendon autograft. Measurements were performed with two force plates. The lower limb symmetry index (LSI) was calculated to determine whether a side-to-side leg difference was classified as normal (LSI >90%) or abnormal (LSI <90%).

**Results:**

The LSI presented high values (>90%) at almost all times before and after ACLR in gait, sprint and single-leg hop tests (*p* < 0.005), with a tendency to increase postoperatively. A lower LSI was observed (<90%) in tests where both extremities were tested simultaneously, such as the drop vertical jump and vertical hop tests (*p* < 0.05).

**Conclusion:**

We observed a tendency to increase symmetry restoration in the kinetics of the involved and uninvolved limb up to twelve months after ACLR, especially in those tests, in which, both limbs were tested individually (gait analysis, sprint and single-leg hop tests). Therefore, the isolation of the involved and uninvolved limb seems to be a critical component in the functional rehabilitation and evaluation of patients before and after ACLR.

**Level of evidence:**

level III.

## Introduction

Anterior cruciate ligament (ACL) injuries commonly lead to abnormal kinematics, kinetics, and muscle activity of the injured extremity. For that reason, it has been suggested that knee function should be examined and considered in the decision making process for ACL reconstruction (ACLR) [1, 2]. Including functional assessments in the evaluation of patients after ACL injury increases our ability to decide who should later undergo ACLR (non-copers) and who may benefit from non-operative management (copers) [3]. These performance-based measures are also important indicators of knee function after ACLR [4, 5].

Knee instability in ACL-deficient individuals has traditionally been assessed using static measures; however, knee instability during dynamic activities is not related to passive measures [6]. Different gait adaptations have been observed in non-copers (individuals who experience knee instability after ACL rupture) soon after ACL injury, which seem to be consistent with their movement and muscle activity during jogging [6–8]. Hop tests are performance-based measures used to assess the combination of muscle strength, neuromuscular control, confidence in the limb, and the ability to tolerate loads related to sports-specific activities [9–12]. These tests can detect limb asymmetries in patients before and after ACLR. However, while unilateral deficits are present in patients after ACLR, these may not be evident during activities involving both lower extremities. For this reason, it has been suggested that isolation of the involved limb with unilateral hop tests should be performed to detect discrepancies in function [13]. Previous studies have shown symmetry restoration and functional recovery before and after ACLR after evaluating the hop distances and times of the involved and uninvolved extremity [14, 15]. However, to our knowledge, this is the first study evaluating the kinetics of the injured and non-injured limb (before and after ACLR) during different strenuous activities, ranging from simple walking (gait analysis) to sprint, and different hop tests (including single-leg and bilateral tests).

The purpose of this study was, therefore, to evaluate the functional status prior to and at different times after ACLR, and to analyze the changes in the kinetic patterns of the involved and uninvolved limb lower during gait, sprint and three hop tests.

## Materials and methods

Between January 2007 and May 2009, 105 patients with unilateral ACL injury were recruited for this study. Inclusion criteria were males aged between 20 and 40 years, with a documented and symptomatic ACL injury associated or not to a meniscal tear sustained within the previous three months. Patients were excluded if they presented any concomitant musculoskeletal condition or previous intervention in the lower extremities that could alter the mechanics of the limb (Table 1). All patients were physically active and were able to perform regular daily activities. Before undergoing ACLR, all patients performed a 6-week progressive exercise training program, emphasizing aggressive quadriceps strengthening to restore muscle strength, range of motion and appropriate neuromuscular responses [16].

**Table 1:** Patient demographics

Initial study sample	105 patients
Cartilage lesions	23 patients
Posterior cruciate ligament injury	1 patients
Meniscal tears	5 patients
Medial collateral ligament/lateral collateral ligament injury	2 patients
Final study sample	74 patients
Lost to follow-up	3 patients
Age (years)	34.0 (SD = 9)
Mean weight (Newton)	843.0 (SD = 20.32)
Right knees	46 (62%)
Left knees	28 (38%)
Mean follow-up	12 months

After concluding this rehabilitation program, all patients completed a standard kinetic protocol which was performed the day before the operation. All patients underwent primary unilateral ACLR using a semitendinosus gracilis tendon autograft obtained from the ipsilateral leg. After surgery, all participants followed the same rehabilitation guidelines [16], and they repeated the same kinetic protocol at 3, 6 and 12 months after the operation. Following surgery, all subjects exhibited full range of motion of the knee, none to minimal joint effusion, and none to minimal pain during ambulation. None of the patients reported episodes of the knee ‘giving way’.

**Fig. 1:**
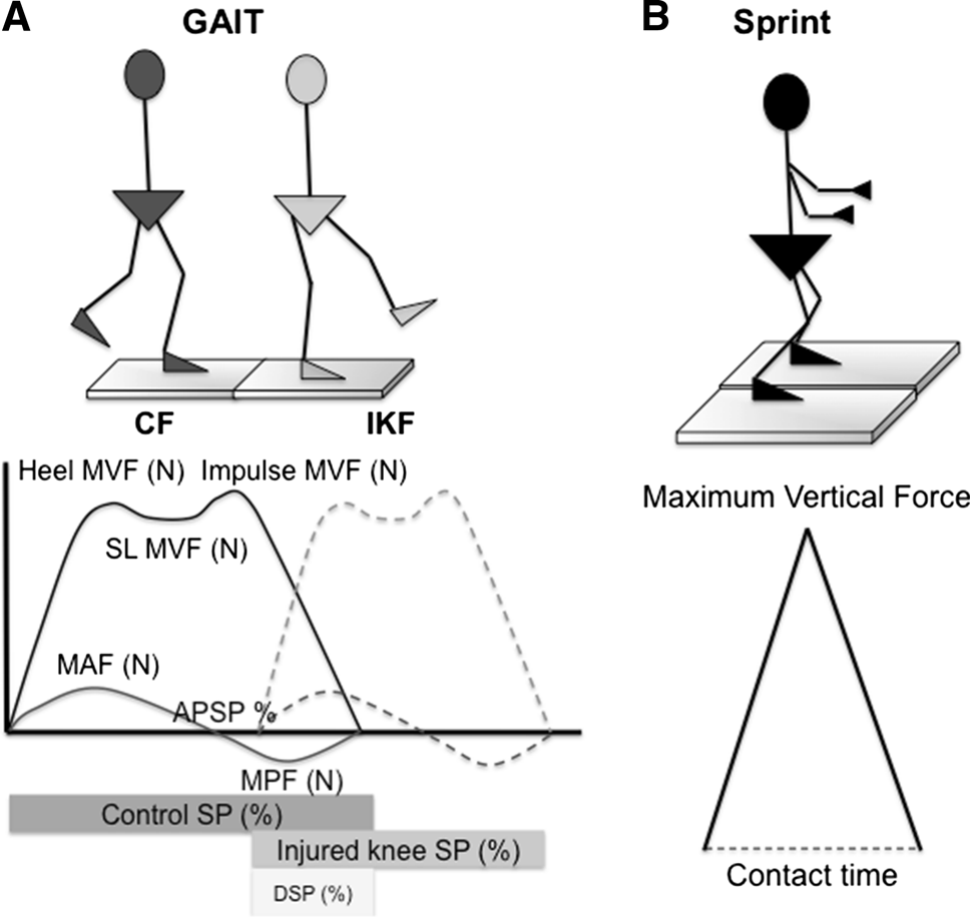
**a** Gait test and kinetics graph. Subjects walked along a 5-m wooden walkway in which one of the force plates was embedded. Subjects were told to walk at a self-selected comfortable pace. **b** Sprint test and kinetics graph. The sprint test was performed with the patient standing on both platforms. After an initial trial, they were instructed to sprint as fast as possible for 5 s (*CF* control foot, *IKF* injured-knee foot, *Max* maximum, *AP* anterior-posterior)

**Fig. 2:**
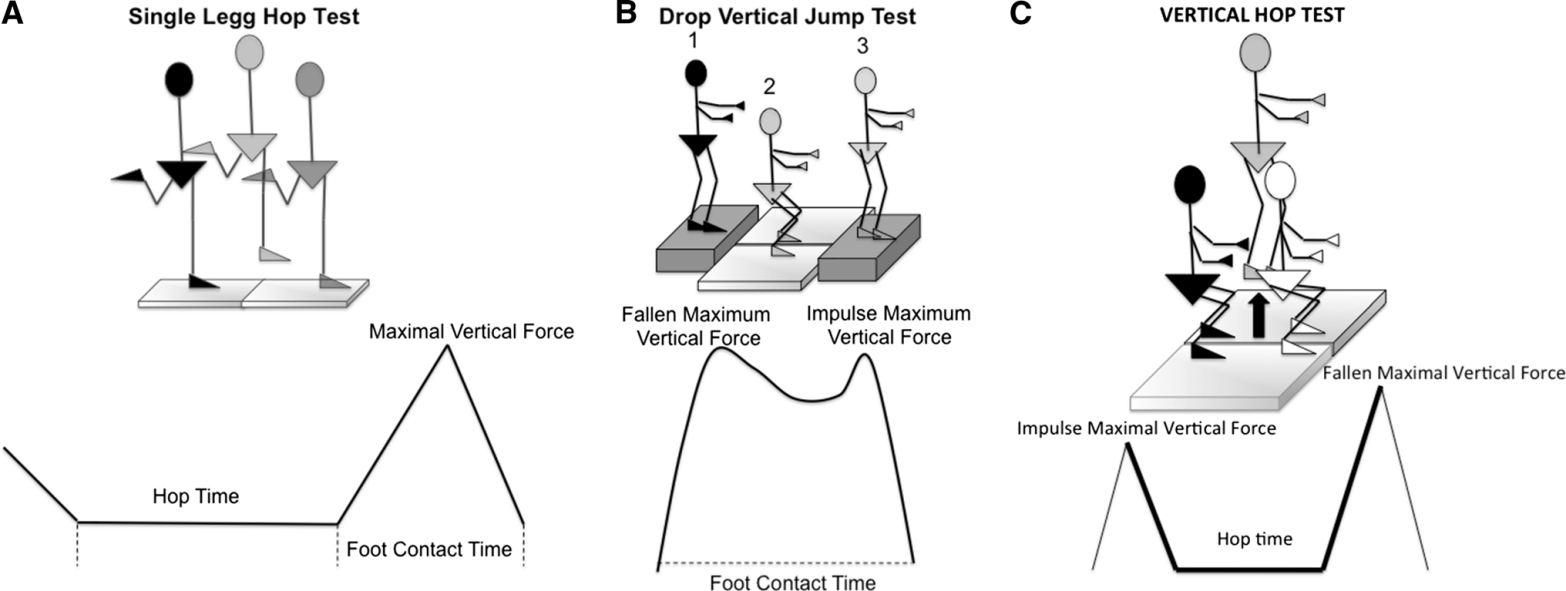
**a** Single-leg hop test and kinetics graph. The single-leg hop test for distance was performed as previously described [18]. Patients were instructed to stand on one leg and to position their toes against a mark on the floor. They were then instructed to hop forward as far as possible and to land on the same leg. **b** Drop vertical jump and kinetics graph. Subjects were instructed to drop off a 30-cm box and perform a maximum jump after landing. The box distance was adjusted so that the patient could land with one foot on each platform. **c** Vertical hop test and kinetics graph. Vertical hop test was performed (Fig. 2c) with the patient standing on both platforms and being instructed to hop using his arms as countermovement. *Max* maximum

**Fig. 3:**
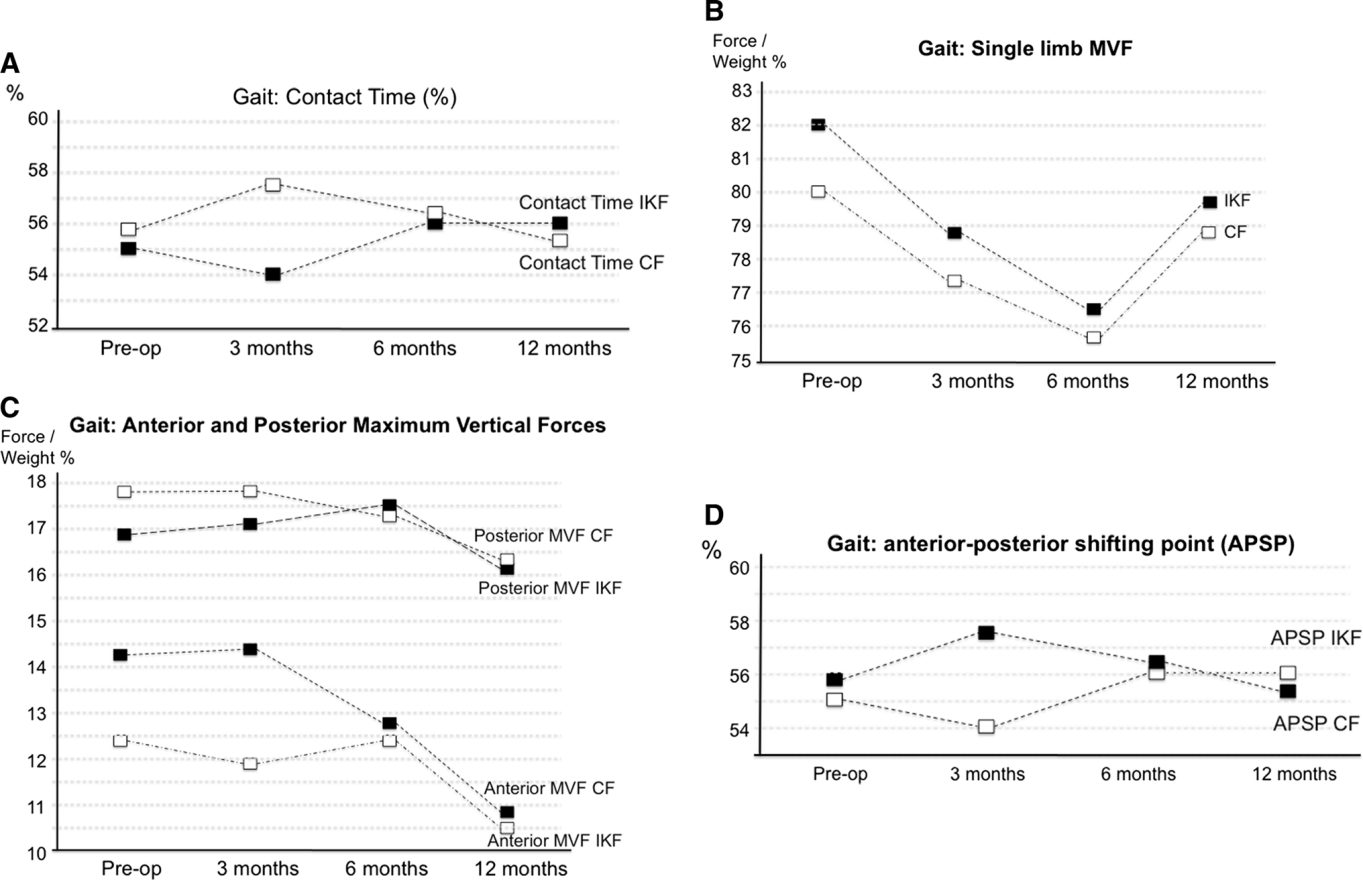
Gait kinetics, **a** contact time, **b** single-leg stance vertical force, **c** maximum anterior and posterior forces, **d** anterior posterior shifting point. *IKN* injured-knee foot, *CF* control foot, *Pre-op* preoperatively

**Fig. 4:**
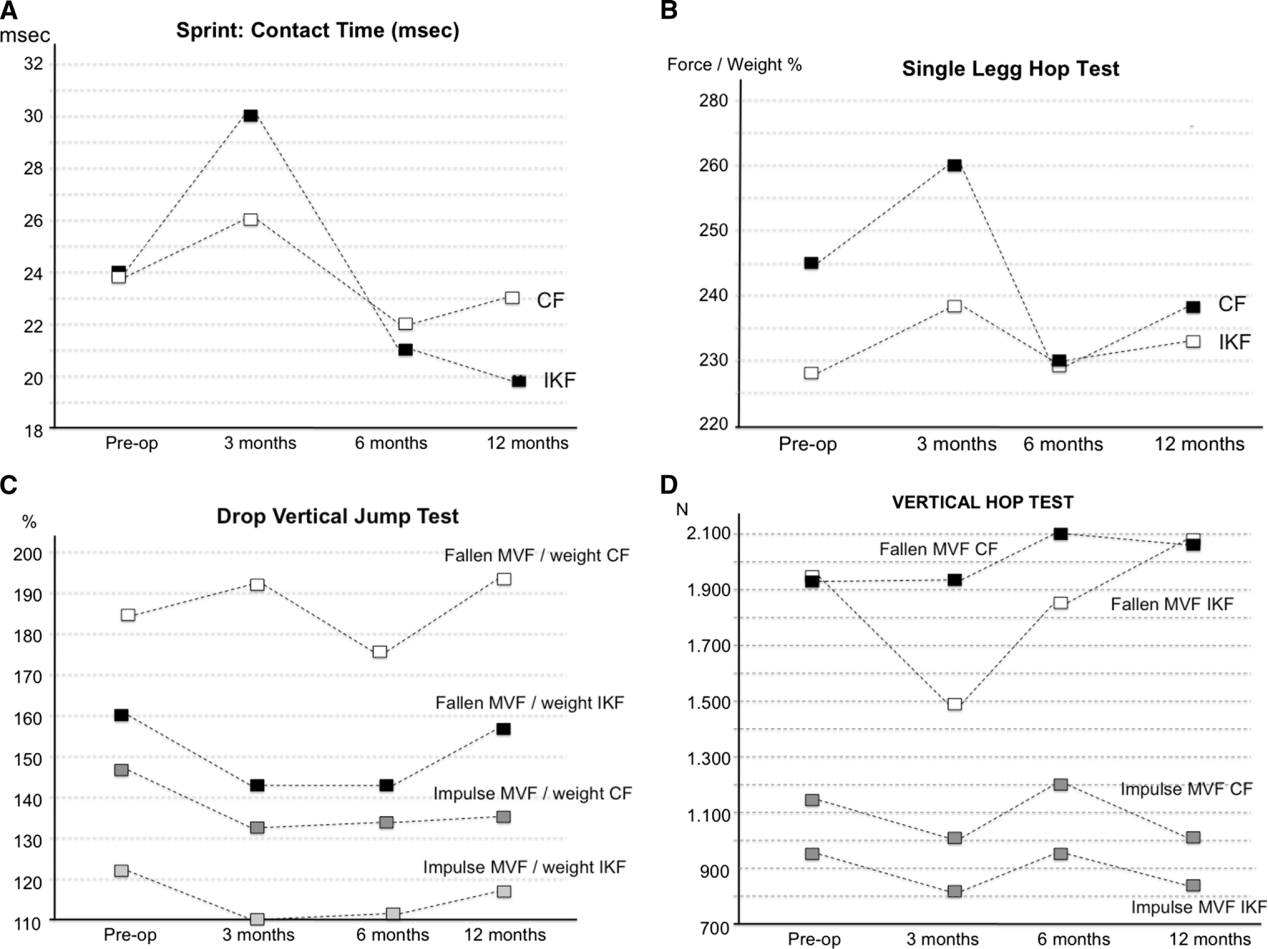
**a** Sprint kinetics, **b** single-leg hop test kinetics, **c** drop vertical jump kinetics, **d** vertical hop test kinetics. *IKF* injured-knee foot, *CF* control foot, *Pre-op* preoperatively

The kinetic protocol included gait analysis, sprint and hop tests (single-leg hop test, drop vertical jump and vertical hop test) (Figs. 1, 2, 3, 4) [17]. All measurements were performed with the use of two Kistler force plates (Kistler^®^; Winterthur, Switzerland) measuring 60 × 90 cm, fixed onto the floor in front of each other. Parameters obtained during gait for the control foot (CF) and injured knee-foot (IKF) included (Fig. 1a) step percentage (SP), double-limb step percentage (DSP), anterior-posterior shifting point (APSP) contact time (CT), heel maximum vertical force (MVF), single-limb (SL) MVF, impulse MVF, maximum anterior force (MAF) and maximum posterior force (MPF). Sprint test parameters included (Fig. 1b) MVF and CT. Parameters obtained from the single-leg hop test included (Fig. 2a) hop time, MVF and CT. Drop vertical jump parameters included (Fig. 2b) fallen MVF, CT and impulse MVF. Vertical hop test parameters included (Fig. 2c) impulse MVF, hop time and fallen MVF. All parameters were normalized by body weight. The lower limb symmetry index (LSI) was calculated to determine whether a side-to-side leg difference was classified as normal (>90%) or abnormal (<90%) [18]. The LSI was defined as the ratio of the involved limb score and the uninvolved limb score expressed in percentage (involved/uninvolved × 100 = LSI). Although LSI scores were the outcome measures of most interest, absolute scores on each lower extremity were also presented for a better understanding of the calculated index score (Tables 2, 3, 4, 5, 6). Patients were carefully instructed on how to conduct each trial before the definitive test was performed (Figs. 1, 2). Data were reviewed for completeness after each trial, and data collection continued until a minimum of three trials were recorded for both limbs. The hop tests were considered valid if the landing was stable. The timing of the kinetic profiles was normalized as a percentage of a single complete cycle.

**Table 2:** Gait kinetics

	Preoperative		3 months		6 months		12 months	
	*X*	SD	*X*	SD	*X*	SD	*X*	SD
MVF heel								
IKF	95.51	1.01	93.39	2.70	92.27	2.58	92.88	2.71
CF	99.81	1.58	98.93	2.19	94.88	3.15	95.46	2.25
*p*	0.001		0.061		0.512		0.312	
LSI	95.6%		94.4%		97.2%		97.2%	
Single–leg MVF								
IKF	82.17	1.3	78.94	2.86	76.47	2.75	79.92	1.5
CF	80.15	1.28	77.23	2.81	75.88	1.68	78.99	2.34
*p*	0.001		0.050		0.0561		0.061	
LSI	102%		102%		100%		101%	
IVF (%)								
IKF	96.93	1.57	95.49	3.18	93.34	2.31	94.69	2.66
CF	100.39	1.58	95.81	2.76	90.13	4.97	93.69	3.37
*p*	0.001		0.222		0.061		0.712	
LSI	96%		99.6%		103%		101%	
Anterior force								
IKF	12.39	4.25	11.58	5.31	12.34	5.7	10.54	4.57
CF	14.36	1.58	14.42	3.61	12.78	4.43	10.80	4.10
*p*	0.001		0.069		0.332		0.077	
LSI	86.2%		80.3%		96.5%		97.5%	
Posterior force								
IKF	16.95	0.63	17.06	1.30	17.51	0.84	16.05	0.6
CF	17.71	0.52	17.75	0.95	17.15	0.97	16.29	1.21
*p*	0.01		0.073		0.0912		0.057	
LSI	95.7%		96.1%		102%		98.5%	
Contact time (%)								
IKF	55.72	0.45	54.29	0.69	56.74	1.66	56.24	0.96
CF	55.85	0.39	57.50	0.88	56.14	1.05	55.30	0.53
*p*	0.069		0.0012		0.067		0.078	
LSI	99.7%		94.4%		101%		101%	

*MVF* maximum vertical force, *IVF* impulse vertical force, *AP* anterior-posterior, *IKF* injured-knee foot, *CF* control foot, *SD* standard deviation

**Table 3:** Sprint kinetics

	Preoperative		3 months		6 months		12 months	
	*X*	SD	*X*	SD	*X*	SD	*X*	SD
MVF								
IKF	180.5	38.4	157.4	37.5	168.6	41.4	180.6	31.4
CF	190.2	32.7	193.8	27.5	183.9	24.0	184.1	29.9
*p*	0.052		0.521		0.067		0.078	
LSI	94.9%		81.2%		91.6%		98%	
Contact time								
IKF	0.24	0.13	0.28	0.19	0.22	0.08	0.18	0.06
CF	0.24	0.13	0.25	0.20	0.21	0.09	0.25	0.20
*p*	0.067		0.172		0.050		0.101	
LSI	100%		112%		104%		72%	

*MVF* maximum vertical force, *IKF* injured-knee foot, *CF* control foot, *SD* standard deviation

**Table 4:** Single-leg hop test kinetics

	Preoperative		3 months		6 months		12 months	
	*X*	SD	*X*	SD	*X*	SD	*X*	SD
MVF								
IKF	228.4	66.9	238.8	42.7	230.6	42.4	233.6	26.85
CF	245.7	45.1	260.6	46.6	229.6	42.8	237.8	58.56
*p*	0.035		0.001		0.001		0.015	
LSI	92.9%		91.6%		100%		98.2%	
Hop time								
IKF	0.18	0.11	0.17	0.04	0.14	0.09	0.22	0.21
CF	0.18	0.09	0.17	0.07	0.11	0.06	0.23	0.24
*p*	0.324		0.823		0.051		0.823	
LSI	100%		100%		127%		95%	
CT								
IKF	0.44	0.19	0.38	0.1	0.38	0.1	0.37	0.11
CF	0.38	0.14	0.39	0.14	0.37	0.13	0.38	0.09
*p*	0.005		0.081		0.143		0.071	
LSI	115%		97.4%		102.7%		97.3%	
CT/hop time								
IKF	0.56	0.9	0.5	0.3	0.42	0.4	0.61	0.5
CF	0.54	0.4	0.45	0.2	0.33	0.18	0.63	0.4
*p*	0.044		0.051		0.026		0.007	
LSI	103%		111%		127%		96.8%	

*MVF* maximum vertical force, *IKF* injured-knee foot, *CF* control foot, *CT* contact time, *SD* standard deviation

**Table 5:** Drop vertical jump kinetics

	Preoperative		3 months		6 months		12 months	
	*X*	SD	*X*	SD	*X*	SD	*X*	SD
FVF								
IKF	160.1	6.31	144.6	15.7	143.1	7.96	157.5	8.27
CF	184.66	6.5	191.5	14.2	176.9	10.1	193.5	13.2
*p*	0.003		0.001		0.054		0.002	
LSI	86.6%		75%		80%		81.3%	
IVF								
IKF	121.4	5.36	106.5	14.2	111.9	6.82	117.4	6.25
CF	146.6	5.14	133.6	12.3	134.9	7.96	135.4	10.4
*p*	0.001		0.043		0.027		0.007	
LSI	82.8%		79.7%		82.9%		86.7%	
CT								
IKF	0.63	0.06	0.46	0.05	0.53	0.05	0.64	0.14
CF	0.62	0.05	0.5	0.05	0.52	0.05	0.65	0.13
*p*	0.567		0.154		0.061		0.077	
LSI	101%		92%		101%		98.4%	

*FVF* fallen vertical force, *IVF* impulse vertical force, *IKF* injured-knee foot, *CF* control foot, *SD* standard deviation, *CT* contact time

**Table 6:** Vertical hop test kinetics

	Preoperative		3 months		6 months		12 months	
	*X*	SD	*X*	SD	*X*	SD	*X*	SD
IVF								
IKF	113.4	25.8	102.5	23.6	107.0	38.2	100.2	12.56
CF	136.6	36.3	127.7	30.2	134.8	32.5	120.5	15.89
*p*	0.035		0.001		0.001		0.015	
LSI	83%		80%		79.3%		83.1%	
FVF								
IKF	233.5	88.9	185.2	71.8	210.1	60.7	250.3	64.08
CF	234.0	67.6	239.5	63.3	236.2	50.7	243.6	31.13
*p*	0.035		0.080		0.200		0.063	
LSI	99.7%		77.3%		88.9%		102%	
IVF/FVF								
IKF	59.93	48.3	60.16	18.9	55.29	27.1	42.43	12.02
CF	64.09	30.6	55.39	13.9	59.94	24.9	50.04	7.83
*p*	0.035		0.432		0.587		0.156	
LSI	93.5%		108.6%		92.2%		84.5%	
Hop time								
IKF	0.42	0.11	0.46	0.11	0.48	0.07	0.47	0.04
CF	0.41	0.11	0.53	0.22	0.47	0.07	0.39	0.14
*p*	0.057		0.762		0.052		0.062	
LSI	102%		86.7%		102%		120%	

*IVF* impulse vertical force, *FVF* fallen vertical force, *MVF* maximum vertical force, *IKF* injured-knee foot, *CF* control foot, *SD* standard deviation

Descriptive statistics, including mean and standard deviation, were used to describe patient demographics. Mean kinetic values at baseline and at 3, 6, and 12 months postoperatively were compared using repeated-measures analyses of variance (ANOVA). For each ANOVA in a significant F ratio, post hoc analysis was performed using *t* test with Bonferroni correction for multiple comparisons; this was performed in order to look at the individual effect rather than the effect of all variables together. All statistical analyses were performed using SPSS v.17.0 for Windows (Chicago, IL, USA). Statistical significance was set as *p* < 0.05.

## Results

Results of gait kinetics are shown in Table 2 (Figs. 1, 2). Although the LSI improved 12 months after surgery for most of the measurements performed, these differences were not statistically significant. The only significant difference was the preoperative and 12-month anterior force; however, this difference was not statistically significant (*p* 0.077). Contact times showed no differences pre- and postoperatively. The sprint kinetics results (Table 3) presented a similar pattern; however, a slight improvement in LSI was observed 12 months after ACLR (*p* 0.078). Single-leg hop test kinetics (Table 4; Fig. 4b) presented a significant improvement in LSI 6 months (100%) after ACLR, which persisted up to 12 months postoperatively (98.2%) (*p* 0.001–0.015). However, drop vertical jump results (Table 5, Fig. 4c) presented a different pattern with a lower LSI 12 months after surgery (*p* 0.002) (<90% at all times). Vertical hop test kinetics showed no differences between preoperative and postoperative LSI values (<90% at all times) (Table 6). Contact/hop times showed no differences preoperatively or postoperatively in all test performed.

## Discussion

The most significant finding of this study is that limb to limb kinetic asymmetries presented a tendency to decrease with time after ACLR in the gait, sprint and single-leg hop tests, with the LSI >90% before and after ACLR. The drop vertical jump and vertical hop tests, however, did not present such behavior with the LSI <90% before and after ACLR.

Our results seem to be consistent with those reported by other authors [14, 15], showing symmetry restoration and functional recovery before and after ACLR in gait, sprint and single-leg hop tests. However, we were not able to observe this phenomenon in all tests performed, since both the drop vertical jump and the vertical hop test did not improve their LSI after ACLR. Logerstedt et al. [15] evaluated functional recovery (quadriceps strength testing, hop testing, and self-reported questionnaires for knee function) in eighty-three athletes after an ACL injury, and at different times after ACLR. They concluded that limb to limb asymmetries are reduced, and normal limb symmetry is returned to a similar level 6 months after ACLR. More recently, Rohman et al. [14] also evaluated changes in the involved and uninvolved limb function after ACLR in 122 patients, with twelve individual tests. From the twelve functional tests in the study, the single-leg squat, retro step-up, single-leg hop, crossover triple hop, and timed hop were suggested to be highly useful tests, since all showed an initial LSI <90%, with significant improvement after rehabilitation. To our knowledge, our study is the first to evaluate LSI functional kinetics in patients before and after ACLR. We included gait, sprint and different hop tests in order to find out if more demanding tests would show any differences. However, we observed that those tests in which the involved and uninvolved leg were tested individually (gait, spring and single-leg hop test) presented a high LSI (>90%) before ACLR, with a tendency to increase at latest follow-up (close to 100%). Nevertheless, those tests in which both legs were tested at the same time (drop vertical jump and vertical hop test) presented a low LSI preoperatively and at all times postoperatively.

Patient management after ACL injury in active individuals may be improved by evaluating function as a consequence of dynamic knee stability using simple hop tests and validated knee outcome surveys, rather than the magnitude of knee laxity and preinjury activity level [19, 20]. Clinicians have traditionally used single-leg hop tests to assess both the patient’s lower extremity muscular strength and the ability to perform tasks that challenge the stability of the knee [21, 22]. For that reason, single-leg hop tests are now commonly used in knee rehabilitation programs. Noyes et al. [23] were one of the first authors to describe a combination of hop tests that mimic the demands of dynamic knee stability during highly demanding activities, and are intended to prepare the patient for a return to such activities [24]. Posteriorly, Gustavsson et al. [25] reported high test–retest reliability, sensitivity, and accuracy after combining three hop tests, that included vertical jump, hop test for distance and hop test performance while developing fatigue (the side hop). More recently, single-leg hop tests have been used to detect persistent limb asymmetries in performance during high-demanding activities, using the lower symmetry index to evaluate the performance between the involved and uninvolved limb [14, 15]. This is preferable to the use of single-limb performance variables because both patients differ in ability, and because (in biomechanical testing) limb symmetry is associated with better rates of return to sports and lower rates of reinjury [24, 26]. Moreover, the current bibliography supports the use of LSI thresholds ranging from 80−90% before recommending return to sports [24, 27, 28]. Nevertheless, the effects of postoperative rehabilitation on the uninvolved limb are not well understood in regard to functional testing. It has been suggested that differences in postural stability after ACLR may be explained by the specific nature of the exercise, and by a possible compensation of the uninvolved lower extremity [21, 29, 30]. Therefore, while unilateral deficits are present in patients after ACLR, these may not be evident during activities involving both lower extremities. For this reason, it has been suggested that the isolation of the involved limb with unilateral hop tests should be performed to detect discrepancies in function [13]. This phenomenon which is not yet well understood, and presents inconclusive data in the literature, may explain the fact that in our study the LSI never improved in tests in which both the involved and uninvolved limb were tested at the same time.

This study presents some limitations. The results can only be generalized to subjects who present with isolated ACL injury, and should not be generalized to individuals with complex concomitant injuries. In addition, as the aim of the study was to evaluate kinetics symmetry (involved and uninvolved limb) restoration before and after ACLR, a comparison group (control group) was not included. Lastly, we did not include any self-reported questionnaires or scores for knee function, which would have added valuable information to the study.

The findings of this study showed a tendency to increase symmetry restoration in the kinetics of the involved and uninvolved limb up to twelve months after ACLR, especially in those tests in which both limbs were tested individually (gait analysis, sprint and single-leg hop tests) as opposed to those tests in which both limbs were tested simultaneously (drop vertical jump and vertical hop test). Therefore, the isolation of the involved and involved limb seems to be a critical component in the functional rehabilitation and evaluation before and after ACLR, as the uninjured contralateral extremity may tend to compensate in activities where both limbs are under stress at the same time, thus diminishing symmetry restoration.
